# The shape of chromatin: insights from computational recognition of geometric patterns in Hi-C data

**DOI:** 10.1093/bib/bbad302

**Published:** 2023-08-28

**Authors:** Andrea Raffo, Jonas Paulsen

**Affiliations:** Department of Biosciences, University of Oslo, 0316 Oslo, Norway; Department of Biosciences, University of Oslo, 0316 Oslo, Norway; Centre for Bioinformatics, Department of Informatics, University of Oslo, 0316 Oslo, Norway

**Keywords:** 3D genome organization, Hi-C, geometric patterns, pattern recognition

## Abstract

The three-dimensional organization of chromatin plays a crucial role in gene regulation and cellular processes like deoxyribonucleic acid (DNA) transcription, replication and repair. Hi-C and related techniques provide detailed views of spatial proximities within the nucleus. However, data analysis is challenging partially due to a lack of well-defined, underpinning mathematical frameworks. Recently, recognizing and analyzing geometric patterns in Hi-C data has emerged as a powerful approach. This review provides a summary of algorithms for automatic recognition and analysis of geometric patterns in Hi-C data and their correspondence with chromatin structure. We classify existing algorithms on the basis of the data representation and pattern recognition paradigm they make use of. Finally, we outline some of the challenges ahead and promising future directions.

## INTRODUCTION

Eukaryotic genomes must be compactly folded and highly organized within the nucleus to maintain cell homeostasis. Spatial proximity of specific genomic loci has been increasingly investigated over the last decades, in particular due to the development of chromosome conformation capture (3C) techniques. Since its inception, the 3C paradigm has been generalized and extended in multiple directions, giving rise to the rapidly-expanding family that is eponymously referred to as *3C-based*. The family includes one-versus-many (e.g. 4C [1]), many-versus-many (e.g. 5C [2]), Capture-C [3], Capture Hi-C [4]) and all-versus-all (e.g. Hi-C [5] and Micro-C [6]) assays. While imaging techniques can spatially localize chromatin loci and thus directly apply geometric analyzes, 3C-based experiments disclose complementary information as spatial proximity frequencies between loci. 3C-based methods are generally based on common experimental steps that include cross-linking with a fixative agent (e.g. formaldehyde), digestion (e.g. by restriction enzymes (REs) or micrococcal nuclease), in-situ proximity ligation, reverse cross-linking and deep sequencing.

## Hi-C DATA IN A NUTSHELL

Hi-C data provide information about the 3D organization of chromatin by measuring the frequency of interactions between proximal pairs of genomic regions, which are typically represented as bins or segments of equal size along the genome. The choice of resolution (i.e. bin size) impacts massively downstream Hi-C data analysis and involves a balancing between sensitivity/sparsity and specificity in the data [7]. In principle, the resolution is only limited by the REs used in the assay, and fragment sizes range averagely from 434 bp (for a four-cutter such as MboI) to 3.7 kb (for a six-cutter such as HindIII). However, high resolution demands sufficient sequencing depth, as it increases by the square of the number of bins [8, 9]. Due to the computational burden of the intrinsic high-dimensionality of the problem, efficient formats have been developed to handle the increased scale of the data at stake [10].

Mathematically speaking, Hi-C data can be represented and interpreted in terms of:

(i) *Matrices*, i.e. rectangular arrays of numbers endowed with a number of mathematical operations. Hi-C data can be stored in a fixed-size symmetrical square table which — at least before further processing — is integer and nonnegative. The adoption of the usual matrix sum and scalar multiplication gives rise to the algebraic structure called vector (or linear) space. Notably, this algebraic interpretation is crucial to exploit methodologies such as Singular Value Decomposition (SVD) and spectral analysis.
(ii) *Images* are scalar functions which, when a resolution is specified, can be sampled over regular 2D grid and stored into a specific matrix; note that the same image can be sampled at different resolutions, resulting in visually distinct outcomes. Although the terms ‘image’ and ‘matrix’ are sometimes used interchangeably, the processing applied to images aims to emphasize visual patterns rather than solely numerical properties: mathematically, geometry is emphasized at the expense of the algebraic structure. In this regard, edges of an image are significantly large local changes in the applicate, i.e. the intensity. On the contrary, objects in images have generally a lower variability in the intensity. When representing Hi-C data as images, a pixel represents a pair of genomic loci and its intensity is, up to a range scaling, the interaction count of such a pair.
(iii) *Weighted graphs*, i.e. a structure amounting to a set of vertices (here: genomic segments) in which some pairs (e.g. those having nonzero interaction frequencies) are connected by edges associated with a scalar (the aforementioned interaction frequencies). As Hi-C matrices are symmetric, the corresponding graph can be considered undirected. Probabilistic graphical models are, theoretically speaking, an extension of graphs that assumes nodes to be random variables, thus allowing to express conditional dependence structures.

Like all experimental techniques, chromosome conformation capture technologies have experimental noise and bias limitations which need to be taken into account in the analysis. Substantial bioinformatics efforts are required to extract reliable contact information. A main source of noise is the presence of miscellaneous undesired linear-type DNAs during proximity-ligation resulting in dangling ends, internal fragments or re-ligation DNA fragments. Random ligations are generally not informative as they can link regions independently of the underlying 3D organization. Noise is potentially further worsened by PCR amplification [11]. Another factor to take into account is related to the genomic distance effect, namely the tendency of higher prevalence of crosslinks between genomic loci close together along the genome even in the absence of any specific higher-order structure [12]. To mitigate the various biases that might be present while possibly enhancing patterns, Hi-C data are often pre-processed with procedures that depend on the data representation used (e.g. via the Iterative Correction and Eigenvector decomposition, ICE, for Hi-C matrices); as a result, the resulting transformed Hi-C data are not necessarily integer or positive [10].

## GEOMETRY ENTERS IN Hi-C

Recently, geometry has established itself as an integral part of Hi-C data analysis as more and more geometric shapes (including points, segments, squares, etc.) are being discovered. In Hi-C data, identifying geometric structures is assumed to correspond to inferring chromosome structural features from a biological perspective, which is one of the major goals in Hi-C data analysis [13]. Despite the progress made through the years, the recognition of patterns in contact maps remains challenging for multiple reasons. First and foremost, formal definitions of such families of patterns are missing: patterns are (usually) not defined explicitly, but rather as the output of methods that are often intended for other uses (e.g. the search for checkerboard-like patterns via principal component analysis PCA), despite their blatant geometric nature. Secondly, contact maps exhibit more than one pattern at a time: these can have different shapes and are potentially overlapping, making it necessary to find ways to decompose them into primitive elements. Due to the aforementioned lack of formal definitions for these patterns, distinguishing between different types of interactions can be difficult. Current approaches often analyze each pattern separately, by assuming that either the effect of other patterns is negligible or that the other patterns can be normalized out of the data. Lastly, Hi-C data are drawn from a population of cells, meaning that patterns in it will not be necessarily present in individual cells or subpopulations; to this end, recent efforts have focused on the estimation of cell type composition from Hi-C data, e.g. by using statistical deconvolution methods [14].

**Figure 1:**
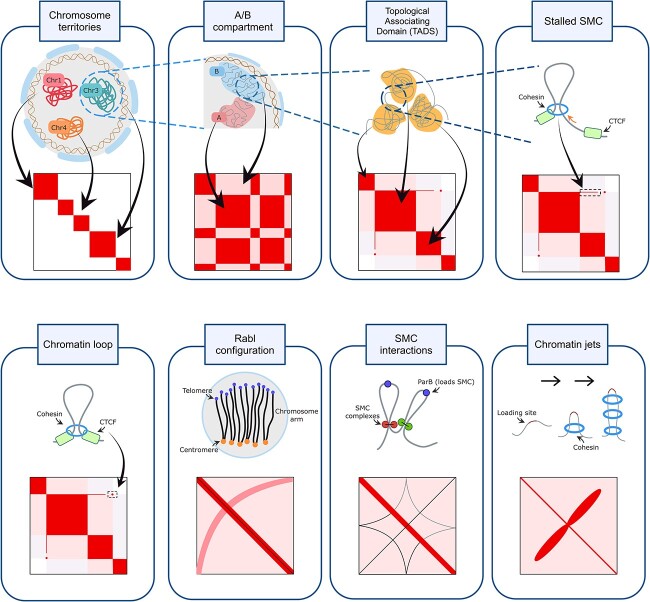
Biological structures and their geometric counterparts in Hi-C data. This graphical representation was adapted from similar ones found in [34, 35]. A tabular representation can be found in Table 1.

The remaining of this section is organized as follows. We start by focusing on three families of geometric shapes typically recognized in Hi-C data, reporting their average size in mammals: squares and rectangles, points and segments. We then briefly discuss more complex patterns that have been observed in contact maps, but for which no recognition algorithm has been proposed yet. For each geometric pattern, the biological processes known to be among its causes are discussed, thus outlining a correspondence between biology and geometry (see also Table 1 and Figure 1).

**Table 1:** Biological processes and corresponding geometric patterns found in Hi-C maps. A graphical illustration can be found in Figure 1

Biological structures	Geometric patterns
A/B compartments, subcompartments	Squares and rectangles
TADs, meta-TADs, sub-TADs,	
Structural variations	
Chromatin loops	Points
SMC stalled on one side	Segments
Rabl configurations	Arcs
Chromatin jets	Cassinian ovals
SMC interactions	Astroids

### Squares and rectangles

They are the most common patterns found in Hi-C data, having been observed first in [5] as ‘large blocks of enriched and depleted interactions, generating a plaid pattern’.

The highest organization level in the interphase nucleus corresponds to that of chromosome territories (CTs), discrete regions with distinct nuclear positions and different gene densities. While their existence was first suggested in 1885 by Carl Rabl [15] for animal cell nuclei, it was only in the 1980s that the concept was ultimately confirmed by the development of the fluorescence in situ hybridization (FISH) technique. In Hi-C data, CTs appear as non-overlapping squares (corresponding to each chromosome) placed along the diagonal.

At megabase resolution, chromosomes appear to be segregated into two major compartments, A and B. Compartments A appear to be more accessible to DNase I, more gene-rich and contain chromatin that is more open and active than their counterparts — compartments B. Geometrically, a plaid or checkerboard pattern — which consists of a partition into rectangles with no internal T-junction — can be observed for both intra- and interchromosomal Hi-C contact maps; in geometric modeling, such split comes under the name of tensor-product mesh. It was later discovered that A/B compartments divide into subcompartments, each bearing a distinctive pattern of genomic and epigenetic features such as gene expression, active and repressive histone marks, DNA replication timing and specific subnuclear structures [16].

Descending in size, topologically associating domains (TADs) take the form of diagonally-placed squares with sides between hundreds of kilobases to a few megabases. TADs are characterized by preferential intra-domain interactions compared to inter-domain interactions with neighboring domains. Sequences within a TAD harbour distinct histone chromatin signatures, expression levels, DNA replication timing, lamina association and chromocenter association [17]. The silencing of repressed developmental genes was linked to long-range TAD-TAD interactions that form constitutive and variable TAD cliques [18]. Unlike the larger-scale A and B compartments, TADs do not necessarily produce checkerboard patterns in 2D contact matrices. It was suggested the existence of higher- and lower-order structures named meta-TADs and sub-TADs, with the former being aggregates of proximal TADs in a genomic neighborhood while the latter being split into regions that display more localized contacts [19].

Genomes can also harbor structural variations (SVs), including translocations or copy number alterations. In Hi-C data, such alterations typically give rise to single- or paired- rectangles with strong chromatin interaction signal at one of the vertices [20]. Although these patterns are (in theory) geometrically simple, their detection is further complicated by two main challenges: the local variation in signal and the fact that basic shapes can be combined or overlaid to form more intricate patterns, such as paired-rectangles.

### Points

Another organization level that was recently described is that of chromatin loops — pairs of genomic loci lying on the same chromosome, despite lying linearly far apart [21]. However, this simple definition does not incorporate the required genomic length of such stretches or the degree of proximity. In eukaryotic cells, chromatin loops are known to link elements such as enhancers or transcription factor-binding sites (TFBS), spatially close to their target genes. Most chromatin loops are located within the boundaries of tissue-invariant TADs [22], and are formed by a process called loop extrusion [23, 24, 25]. In Hi-C contact maps, chromatin loops manifest as points, somewhat-circular (blob-shaped) objects with their own specific scale [26].

### Segments

The term architectural stripe is commonly used to indicate interactions between a single locus and a contiguous genomic interval which, biologically, points to structural maintenance of chromosomes (SMC) complexes stalled on one side. Such a structure started catching the attention of researchers only recently [27]. Geometrically, they resemble segments, but are usually referred to as lines, flames or simply stripes by the bioinformatics community. Being the stripe architecture a relatively new observation, a formal closed definition is still missing. Despite having been originally linked to asymmetric loop extrusions at TAD boundaries [25, 28], it was later noted that segments can also appear without a TAD being clearly observed [29].

### Complex patterns

In addition to the geometrically simple patterns discussed in the previous sections, more complex shapes have recently been identified in Hi-C data thanks to the continuous progress in 3C-based technologies.

In plants, the so-called Rabl configuration of interphase nuclei appears like an anti-diagonal pattern which approximates the border of an arc (i.e. a portion of the circumference of a circle). The Rabl configuration is characterized by the attachment of centromeres and telomeres to opposite sides of the nuclear envelope [30]: it is crucial to ensure the orientation of chromosomes in nuclei with the purpose of maintaining chromosomal integrity and aiding the alignment of homologs during meiosis [31].

Another example is that of cohesin-propelled chromatin jets in quiescent mammalian lymphocytes [32], characterized by figure-eight shape patterns that loosely recall flattened lemniscates or Cassinian ovals. Jets propagate symmetrically for 1–2 Mb unless constrained by CTCF, which can convert bi- to unidirectional extrusion or deflect the angle of the jet propagation.

Non-trivial interactions occur between SMC complexes translocating from opposing sites in the Bacillus subtilis chromosome, resulting in a complex shape pattern that is mathematically known under the name of astroid [33].

## GEOMETRIC PATTERN RECOGNITION FROM AFAR

The automatic recognition of patterns and regularities is of paramount importance in applied fields, as it facilitates the description, analysis and comparison of data. The subfield of pattern recognition we are interested in, called geometric pattern recognition, focuses unsurprisingly on the detection and of geometric patterns in input data. Following the classification proposed in various publications of the field (see, for example, [36–38]), the methodologies adopted in Hi-C data analysis can also be divided into four major groups: template-based, structural, statistical and learning-based.

*Template-based pattern recognition* is one of the earliest approaches to pattern recognition, first successfully used in speech recognition and optical character recognition (OCR). It consists in matching (part of) the input data with one or more members of a template while enforcing invariance to classes of transformations. However, it was not the first strategy used in the geometric analysis of Hi-C data. An example of application is the recognition of chromatin loops in terms of a template of dot-like shapes, as we will see for the software MUSTACHE in Section Points. The use of rigid templates allows to re-apply the same method to new data without tedious training, fine-tuning or redesign.

In *structural* or *syntactic pattern recognition*, semantic primitives written in some description language are used to represent some input data, together with a set of rules (the grammar) that defines possible composition relations. This paradigm has been extensively applied in Hi-C data analysis: to give an example, TADs are often defined by first recognizing horizontal and vertical segments, which are then aggregated into squares — and possibly hierarchies of rectangles — on the basis of some criteria (see Section TADs). Syntactic algorithms can result in a combinatorial explosion of possibilities to be investigated.

*Statistical pattern recognition* interprets each pattern in terms of $d$ features, while input data are translated into points of a $d$-dimensional (usually Euclidean) space; such points are then analysed through statistical decision and estimation theories (e.g., kernel methods or Bayesian analysis). Statistical methods place a strong emphasis on inference by constructing and fitting probability models that are specifically customized for the given task. This enables the computation of quantitative measures of confidence such as $P$-values, providing valuable insights into the reliability of the results. Here, the choice of the feature representation and the assumptions on the underlying distributions strongly influence the result. A use case is domainClassifyR’s recognition of segments via $Z-$statistics, see Section Segments.

*Learning-based pattern recognition* assigns existing (supervised learning) or novel categories (unsupervised learning) to input elements with minimal assumptions about the data-generating system. Avoiding model assumptions can be effective for generating predictions dealing with data collected without a meticulously controlled experimental design and in the presence of complex nonlinear interactions; on the other hand, these solutions may lack direct connection to existing biological knowledge despite strong predictions [39]. The identification of A/B compartments is mostly based on PCA — a well-known technique in (unsupervised) dimensionality reduction, see Section Compartments.

In practice, pattern recognition often defies neat categorization, as methods commonly incorporate multiple theoretical paradigms instead of adhering strictly to a single category.

We now discuss existing methods that have been used to detect geometric patterns in Hi-C data, following the same organization of Section Geometry enters in Hi-C. We here classify such approaches based on the representation of Hi-C data (matrix-based, image-based, or graph-based) and the class of pattern recognition methodology (template-based, structural, statistical and learning-based) they make use of.

## SQUARES AND RECTANGLES

### Compartments

The discovery of a plaid pattern that decomposes Hi-C maps into two types of loci (the A and B compartments) was first presented in [5]. In the paper, each chromosome in a genome-wide Hi-C contact map from a karyotypically-normal human GM06990 lymphoblastoid cell line is partitioned by using PCA, a popular learning-based technique for the analysis of data in matrix form. The authors conclude that, for all but two chromosomes, the first principal component can unveil the plaid pattern; for the remaining two chromosomes, the first principal component corresponds to the two chromosome arms, but the second principal component delineates the plaid pattern. Since then, PCA has become one of the de facto standard ingredients to identify compartments, giving rise to the large family of PCA-based (i.e. unsupervised-learning-based) methods. PCA has been implemented in a large number of tools, both in its classical (e.g. Juicer’s eigenvector [40], HOMER’s runHiCpca [41] and HiCdat [42]) and memory-efficient (e.g., POSSUMM [43] and dcHiC [44]) formulations. More precisely, POSSUMM [43] accelerates the computation of the eigenvector decomposition via the power method. On the other hand, dcHiC [44] implements a parallelized partial SVD, thanks to which it is possible to compute just the first few singular vectors (i.e. the eigenvectors) needed for compartment analysis.

Recently, alternative solutions that do not use PCA have been proposed: CscoreTool [45] — which infers compartments via statistical-based modeling of Hi-C matrices — and Calder [46] — that identifies compartment domains by segmenting each chromosome into regions having high intra-region similarity and low inter-region similarity (here, by clustering contact similarities defined in terms of Fisher’s z-transformed correlations – thus combining the statistical and learning-based paradigms).

The main characteristics of these compartment callers are reported in Table 2. Interestingly enough, methods are typically matrix-based and do not rely on template-based or structural pattern recognition.

**Table 2:** Main characteristics of different compartments and subcompartment callers, sorted by publication year

	Caller	Input details	Parameters	Data type	PR family	Description	Complexity	Language
compartments	HOMER	BED file	11	Matrix	UL	Checks the first two principal components (PCs) of the distance-normalized interaction matrix	${\mathcal{O}}(n^3)$	Perl, C++, R
	HiCdat	BAM file	1	Matrix	UL	Analyses the sign of the first PC of the distance-normalized and correlated intra-chromosomal interactions	${\mathcal{O}}(n)$	C++, R
	Juicer	.hic file	4	Matrix	UL	Utilizes the sign of the first PC of the Pearson’s matrix	${\mathcal{O}}(n^3)$	Java
	CscoreTool	BED file	3	Matrix	STAT	Uses C-scores to deduce a log-likelihood function, which is then maximized	NA	C++
	Calder	3-column txt file	5	Matrix	STAT, UL	Clustering contact similarities (Fisher’s z-transformed correlations)	${\mathcal{O}}(n^3)$	R
	POSSUMM	Sparse 2D array	5	Matrix	UL	Accelerates the computation of the eigenvectors via the power method	${\mathcal{O}}(n)$	R
	dcHic	Sparse 2D array	2	Matrix	UL	Exploits a parallelized partial singular value decomposition	NA	Python, R
subcompartments	GaussianHMM	2D array	1	Matrix	STAT	Applies a Gaussian HMM clustering algorithm to find six subcompartments	NA	Python
	SNIPER	.hic file	0	Matrix	UL, SL	Use of an autoencoder NN (feature extraction and dimensionality reduction) and an MLP classifier (subcompartment labeling)	NA	Python
	SCI	BED file	5	Graph	STAT	Combines graph embedding (for dimensionality reduction) with $k$-means clustering (to determine five subcompartments)	NA	Python, C++
	Calder	3-column txt file	5	Matrix	STAT, UL	Divisive hierarchical clustering within each domain to locate eight subcompartments in each compartment	${\mathcal{O}}(n^3)$	R

The parameter $n$ denotes the size of the Hi-C matrix.

The following abbreviations are used for the families of pattern recognition (PR) algorithms: SL = supervised learning, STAT = statistical, UL = unsupervised learning.

### Subcompartments

When it comes to detecting subcompartments, no standard has been established yet. Most approaches base their pipelines on matrix properties. The method in [16] applies a Gaussian Hidden Markov Model clustering algorithm (GaussianHMM) to contact maps from human lymphoblastoid cells. The analysis points to the existence of (at least) six subcompartments (A1-2, B1-4) with distinct patterns of histone modifications. The authors claim that similar results are obtained when using $k$-mean and hierarchical clustering. SNIPER [47] studies Hi-C matrices via neural networks. It divides A/B compartments into the five subcompartments A1-2, B1-3 by subsequently applying two separate neural networks: a denoising autoencoder, which is used to extract features while reducing the dimensionality of the input data, and a multi-layer perceptron (MLP) classifier, used to categorizes the regions into one of five primary subcompartment classes. In Calder [46], a score matrix that aims at summarizing the plaid pattern is computed for each compartment; the score matrix is decomposed via PCA, and the first 10 principal components are used to partition the compartment further through divisive hierarchical clustering. The final step estimates the likelihood of nested subdomains via a mixture log-normal distribution. In its second version, dcHiC [44] finds subcompartments by using a Hidden Markov Model segmentation on the basis of the magnitude of the first principal component.

Recent advancements in the representation of Hi-C data involve the utilization of graphs. One notable method, SCI [48], has emerged, enabling the transformation of the Hi-C interaction graph into a lower-dimensional vector space through graph embedding. Subsequently, SCI employs $k$-means clustering to predict sub-compartments within the data.

Also noteworthy is the the absence of template-based and structural pattern recognition, with all methods relying on statistical or learning-based paradigms and adopting either the matrix or the graph representation of Hi-C data. This point is also visible in Table 2, which summarizes the key attributes of subcompartment callers.

### TADs

Significant progress has been made in the field of TAD detection, with a diverse range of algorithms now available (see Table 3), in contrast to the relatively limited focus on compartments and subcompartments. Initially, TAD callers looked for consecutive diagonally-placed square regions with higher number of interactions. Since the hierarchical structure in TADs was discovered, most of the latest TAD-calling methods have been conceived to identify hierarchies of TADs. The reader is referred to [49–53] for existing reviews, surveys and benchmarking studies.

**Table 3:** Main characteristics of different TAD callers, sorted by publication year

Data type	TAD caller	Input details	Parameters	PR family	Hierarchical?	Complexity	Language
Matrix	DI	2D array	3	STRUCT	Disjoint	NA	MATLAB, Perl
	Armatus	2D array	1	STRUCT	Overlapping	${\mathcal{O}}(tn^2)$	C++
	Arrowhead	.hic file	1	STRUCT	Overlapping	${\mathcal{O}}(n^2)$	Java
	chromoR	2D array	2	STAT	Disjoint	NA	R
	CHDF	2D array	1	UL	Disjoint	NA	C++
	IS	2D array	5	STRUCT	Disjoint	NA	Perl
	TADTree	2D array	6	STAT, STRUCT	Overlapping	${\mathcal{O}}(ns^5)$	Python
	TopDom	2D array	1	STRUCT	Disjoint	NA	R
	CaTCH	sparse 4-column file	1	UL, STRUCT	Nested	NA	R
	ClusterTAD	2D array	1	UL	Disjoint	NA	MATLAB
	GMAP	2D array	10	STAT, STRUCT	Nested	NA	R, C++
	HiTAD	.cool file	1	STRUCT	Nested	NA	Python
	IC-Finder	2D array	2	UL	Disjoint	NA	MATLAB
	PSYCHIC	2D array	1	STAT, STRUCT	Nested	NA	MATLAB
	TADbit	2D array	1	STAT	Disjoint	NA	Python, C
	HiCExplorer	h5 file	4	STAT	Disjoint	NA	Python
	HOMER	BED file	5	STRUCT	Disjoint	NA	Perl, C++
	MSTD	2D array	1	UL	Disjoint	NA	Python
	OnTAD	2D array	5	STRUCT	Nested	${\mathcal{O}}(ms^2)$	C++
	Constrained HAC	2D array	1	UL	Disjoint	${\mathcal{O}}(n(h+\log (n)))$	R
	Matryoshka	2D array	1	UL, STRUCT	Nested	${\mathcal{O}}(tl^2)$	C++
	TADPole	2D array	3	UL, STRUCT	Nested	NA	R
	FrankenTAD	2D array	6	STRUCT	Disjoint	NA	Go
	GRiNCH	2D array	3	UL	Disjoint	${\mathcal{O}}(kn^2)$	C++
	HICKey	2D array	3	STAT, STRUCT	Nested	${\mathcal{O}}(n^3)$	C++
Image	EAST	2D array	3	TMP	Disjoint	${\mathcal{O}}(n^2)$	Python
	CHESS	.hic or cool files	4	STAT, UL	Disjoint	$NA$	Python
	HiCseg	2D array	3	STAT, STRUCT	Disjoint	${\mathcal{O}}(Kn^2)$	R, C
	TADBD	2D array	2	TMP	Disjoint	NA	R
Graph	Spectral	2D array	2	STRUCT	Disjoint	NA	MATLAB
	MrTADFinder	Sparse 3-column file	1	STRUCT	Disjoint	${\mathcal{O}}(n^3)$	Julia
	3DNetMod	Sparse 3-column file	18	STRUCT	Overlapping	${\mathcal{O}}(n)$	Python
	deDoc	Sparse 3-column file	0	STRUCT	Nested	${\mathcal{O}}(n\log ^2n)$	Java
	SpectralTAD	2D array	11	STRUCT	Disjoint	${\mathcal{O}}(n)$	R
	SuperTAD	2D array	0	STRUCT	Nested	${\mathcal{O}}(n^4L^2H)$	C++

The following parameters appear in the column reporting the computational complexity: $n$ is the size of the Hi-C matrix; $t$ is the number of resolutions to be inferred; $s$ is the maximum size of the inferred TAD; $m$ is the expected count of possible boundaries; $h$ identifies the bandwidth; $l$ refers to the interval frequency while clustering the inferred $s$ resolutions; $k$ is the rank of the low-dimensional matrices; $K$ defines the maximum number of diagonal TAD partitions; $L$ denotes the maximum number of leaves, while $H$ denotes the maximum height at which the coding tree is found.

The following abbreviations are used for the families of pattern recognition (PR) algorithms: STAT = statistical, STRUCT = structural, TMP = template-based, UL = unsupervised learning.

#### Matrix representation

The matrix representation of Hi-C data proved enormously popular, with more than twenty methods proposed in the last decade.

Most of the initial approaches adopted the following structural methodology: definition of a score function; extraction of significant local extrema through optimization algorithms, which are assumed to locate potential TAD boundaries; construction of higher order structures (squares) from the candidate boundaries according to some criteria aimed at filtering out false positives. The structural family includes methods that compute: (i) the interaction frequency of the surrounding regions at each locus, e.g. Armatus [54], Insulation Score (IS) [55], TopDom [56] and OnTAD [57]; (ii) the upstream or downstream interaction bias for a genomic region, e.g. Directionality Index (DI) [58] and HiTAD [59]; (iii) other TAD features, e.g. Arrowhead [16], HOMER’s findTADsAndLoops [60] and FrankenTAD [61].

Learning-based pattern recognition has proliferated through hierarchical and partitional clustering. Hierarchical methods construct dendrograms — trees that represent the relationship of similarity among the bins under study — and then proceed by cutting it at a certain level by using some threshold, as for Constrained HAC [62] and TADPole [63]; another example of interest is that of Matryoshka [64], which builds a novel algorithm on top of Armatus. Partitional algorithms produce a partition into a specified number of clusters by either minimizing or maximizing some numerical criteria: in ClusterTAD [65], the criterion is the within-cluster sum of squares for $k$-means clustering; in GRiNCH [66], it is the sum of pairwise dissimilarities for $k$-medoids clustering; in CHDF [67] it is the sum-of-squared error with respect to three kind of regions (domain regions, regions between adjacent domains and the residuals). Clustering-like approaches do not properly make use of cluster analysis but introduce methodologies that are inspired by how clustering work. For example: IC-Finder [68] starts by considering each column as a single cluster, then merges adjacent clusters if a criterion based on two parameters — heterogeneity and local directionality index — holds; MSTD [69] identifies TADs by grouping points in rectangular shapes by first identifying cluster centers as points with an anomalously large local density, and then by associating each point to the closest center; CaTCH [70] partitions the genome into a set of domain seeds of fixed size, which are then progressively merged into larger domains by thresholding a tailor-made metric called reciprocal insulation.

Many methods in the field can be classified as statistical pattern recognition techniques, such as $z$-scores (e.g. HiCExplorer’s hicFindTADs [71]), BIC-penalized likelihood (e.g. TADbit [72] ), generalized likelihood-ratio tests (e.g. HICKey [73]), Poisson distributions (e.g. chromoR [74] and Gaussian Mixture Models (e.g. GMAP [75]). Tailor-made models were also proposed: TADTree [76] defines a model that depends on two parameters – $\beta $, the baseline enrichment for contacts between adjacent bins within the TAD and $\delta $, the rate at which contact frequency increases with distance between bins; PSYCHIC [77] introduces a two-component probabilistic model corresponding to the probability of intra- and inter-TAD interactions.

Note that all methods producing overlapping or nested hierarchical TADs can be also considered as adopting the structural paradigm, as composition relations between square patterns are imposed.

#### Image representation

Even though TAD detection might seem fully rooted in the field of computer graphics, only a few methods interpret Hi-C data in terms of images.

EAST [78] and TADBD [79] use a template-based approach by applying Haar-like features — a set of adjacent rectangular regions, each of which has a certain weight — via the summed-area table data structure.

CHESS [80] offers a pipeline rooted in image processing: (1) denoise the image using a bilateral filter; (2) smooth the image using a median filter; (3) image binarization using Otsu’s method; (4) morphological closing of the image; (5) computation of 2D cross-correlation between all the extracted areas, which are grouped by $k$-means clustering to detect main structural features.

HiCSeg [73] turns the initial 2D segmentation problem into a 1D one by maximum likelihood estimation of three possible distributions: Gaussian (for normalized Hi-C data), Poisson and Negative Binomial (for raw Hi-C data). TAD boundaries are found by maximizing the likelihood via dynamic programming.

#### Graph representation

The weighted graph that originates by interpreting the Hi-C matrix as an adjacency matrix is here decomposed into subgraphs by minimizing or maximizing different measures.

A first case is the Fiedler number, also known as algebraic connectivity in graph theory. Spectral [81] computes its Laplace matrix and extracts the Fiedler number and vector to perform a first split. The process is iterated until the Fiedler number of all sub-matrices is larger than the threshold or the TAD size reaches a pre-set lower bound. SpectralTAD [82] accelerates the application of spectral graph theory used in Spectral by applying sliding windows along the matrix diagonal.

Another measure is that of modularity, which quantifies the strength of a split of a network into communities. MrTADFinder [83] defines the modularity and objective function in a randomized null model for Hi-C maps, then optimizes the objective function with a heuristic algorithm. 3DNetMod [84] maximizes network modularity via a Louvain-like, locally greedy algorithm.

Finally, structural entropy was also considered. deDoc [85] partitions the original weighted undirected graph into subgraphs so that the uncertainty embedded in the dynamics of the graph (i.e. its structural information or entropy) is minimized; the algorithm produces a tree, and TADs are extracted as the continuous leaf nodes in it. SuperTAD [86] finds optimal coding trees from a contact map in a polynomial-time solvable; while using the same paradigm as deDoc, it can return hierarchical TADs with more than two levels.

## SEGMENTS

Differently from compartments, subcompartments and TADs, all available methods introduced for the recognition of segments rely on the image representation of Hi-C data, and borrow existing concepts from computer vision and image processing, see Table 4.

**Table 4:** Main characteristics of different segment callers

Segment caller	Input details	Parameters	PR type	Description	Location	Language
Zebra	.hic files	0	MAN	Manual curation of pixels with high interaction frequency	TAD boundaries	R
domainClassifyR	2D array	1	STAT	TAD recognition followed by computation of stripe score	TAD boundaries	R
CHESS	.hic or cool files	4	STAT, UL	Application of filters, feature extraction and $k$-means	no restriction	Python
Chromosight	cool file	4	TMP	Convolution of template patterns	no restriction	Python
Stripenn	cool file	5	STRUCT	Image preprocessing followed by Canny edge detection and segments recognition via a set of criteria	no restriction	Python

All methods are image-based, and none reports their computational complexity.

The following abbreviations are used for the families of pattern recognition (PR) algorithms: MAN = manual, STAT = statistical, STRUCT = structural, TMP = template-based, UL = unsupervised learning.

Zebra [27] is a manual method, thus not belonging to any specific type of pattern recognition. It searches for pixel tracks of higher interaction frequency at the boundaries of genomic domains, which must then be manually processed to decide which candidates are segments and which ones are not. Zebra lacks a quantitative assessment of segments, and its code is not publicly available. An alternative implementation of this algorithm, made available by an independent group, can be found on GitHub under the name StripeCaller (https://github.com/XiaoTaoWang/StripeCaller).

Statistical pattern recognition includes domainClassifyR [87] and CHESS [80], being the latter described in Section TADs. The approach named domainClassifyR starts marking TADs and then measures their stripe score, a measure based on the $Z$-statistic. Intra-TAD segments remain undetected.

Chromosight [88] works by convolving templates over the contact map, as done in computer vision tasks involving images; thus, the method is clearly template-based. Then, candidates are analyzed and possibly discarded with respect to a set of criteria, i.e. if they overlap too many empty pixels or are too close to another detected pattern.

Stripenn [89] starts by converting the input Hi-C map to a digital image, which is then pre-processed by contrast adjustment and noise reduction. This step is followed by the application of the Canny edge detection algorithm. Vertical lines are then detected and possibly merged, via a set of custom criteria; in this regard, Stripenn can be considered as based on structural pattern recognition. Finally, two coefficients (median $P$-value and stripiness) are computed to evaluate quantitatively architectural stripes.

## POINTS

The identification of strong punctate signals is a critical part of most Hi-C analyzes, as it points to the presence of chromatin loops. Compared to TADs, fewer callers are available and, to the best of our knowledge, no review or survey has been published on the topic. Table 5 summarizes the characteristics of dot callers.

**Table 5:** Main characteristics of different dot callers, ordered by publication year

Data type	Segment caller	Input details	Parameters	PR family	Description	Complexity	Language
Matrix	Fit-Hi-C, FitHiC2	Two $5$-column tables	4	STAT	Spline fitting for initial null model, and estimation of contact probabilities and $P$-values	NA	Python
	HMRFBayesHiC	$4$ -column table	2	STAT	HMRF-based Bayesian method with Ising prior for representing the unobserved peak status	${\mathcal{O}}(n^2)$	R
	GOTHiC	BAM or Bowtie file	2	STAT	Cumulative binomial test to detect loci with higher Hi-C interactions than expected by chance	NA	R
	HiC-DC	$3$ -column table	5	STAT	GLM approach based on zero-truncated negative binomial regression	NA	R
	HOMER	BED file	5	STRUCT	Scoring of locally dense contact regions found in relative contact maps	NA	Perl, C++
	cLoops, cLoops2	BEDPE file	3	UL	cDBSCAN/blockDBSCAN clustering	${\mathcal{O}}(n\log (n))$	Python
	HiCExplorer	cool file	8	STAT	Interaction filtering using negative binomial distribution, followed by comparing candidates to their neighborhoods	NA	Python
	Peakachu	.hic or cool files	0	SL	Searches the best random forest for a two-class problem	NA	Python
	HiC-ATC	TXT file	3	STAT	Makes use of an aggregated Cauchy test to improve the output of existing methods that assume independence in neighboring chromatin interactions	NA	R
	LOOPbit	3-column table	2	SL	CNN that predicts loop locations	NA	Python
	ZipHiC	7-column table	0	STAT	Hidden Markov random field-based Bayesian approach based on a zero-inflated Poisson distribution for noise	NA	R
Image	Juicer	.hic file	0	STAT, UL	Clusters pixels that exhibit significantly higher number of interactions than different neighborhoods	NA	R
	CHESS [80]	.hic or cool files	4	STAT, UL	Application of filters, feature extraction and clustering via $k$-means clustering	NA	Python
	Chromosight	cool file	4	TMP	Convolution of template patterns	NA	Python
	MUSTACHE	.hic or cool file	0	TMP	Computes the scaled normalized-Laplacian of the convolution between the image and Gaussians of increasing scales, followed by an analysis of neighborhoods	NA	Python
	SIP	cool file	9	STRUCT	Image preprocessing, followed by a regional maxima detection algorithm	NA	Java
	LASCA	.hic or cool file	10	STAT	Diagonal filtering of high-intensity pixels via corrected $P$-values, followed by clustering and further filtering	NA	Python
	RefHiC	mcool file	2	SL	Selection of high-intensity pixels via a NN, grouping via density-based clustering and final filtering	NA	Python
Image & graph	GILoop [90]	cool file	0	SL	Dual-branch neural network that learns from both image and graph-representations	NA	Python

The input for HiC-ATC is a txt file from a Hi-C chromatin interaction calling method, such as Fit-Hi-C/FitHiC2.

The following abbreviations are used for the families of pattern recognition (PR) algorithms: SL = supervised learning, STAT = statistical, STRUCT = structural, TMP = template-based, UL = unsupervised learning.

### Matrix representation

Most methods rely on statistical-based modeling. Several computational and statistical methods orbit around the estimation of the expected contact frequencies under the null (i.e. random collisions). Fit-Hi-C [91] fits an initial nonparametric spline using the observed contact counts and genomic distances between all possible mid-range locus pairs; such a spline is used to determine a threshold to identify outliers and exclude them from the calculation of a second spline, which is used to estimate prior contact probabilities for each mid-range locus pair and, subsequently, $P$-values from a binomial distribution. Its latest reimplementation called FitHiC2 [92], allows the user to perform genome-wide analysis for high-resolution Hi-C data, including all intra-chromosomal distances and inter-chromosomal contacts. Another approach within the same paradigm is GOTHiC [93]: it estimates random interaction probability then applies the binomial test to distinguish between random and real interactions. To account for both the zero inflation and over-dispersion of contact counts, HiC-DC [94] performs the estimation of a null or background model via a GLM based on zero-truncated negative binomial regression, which is then employed to assess the statistical significance of unexpectedly large chromatin contacts. Another example of a statistical method using GLM is given by HiCExplorer’s hicDetectLoop [95]: it fits a negative binomial distribution to Hi-C data to filter out interaction pairs with respect to a threshold, then uses a donut algorithm – it considers all elements of the matrix as candidate peaks and compares the region around them to the neighboring interactions. All these methods have the drawback of testing each individual pair of loci independently, ignoring the potential correlation among pairs of loci. To address this point, HMRFBayesHiC [96] considers a hidden Markov random field-based Bayesian method that explicitly models the spatial dependency among adjacent loci. A pseudo-likelihood is used to approximate the Ising distribution, which models the hidden peak status. Due to its heavy computational costs, a modified version of the algorithm that approximates the Ising distribution by a set of independent random variables, allowing a more convenient computation was introduced under the name of FastHiC [97]. An alternative direction is taken by ZipHiC [98], which implements a Bayesian framework to detect enriched contacts. Hi-C data are modeled via a $K$-component mixture density, where the first component is a zero-inflated Poisson (ZIP) distribution for noise, while the other components follow Poisson distributions. Spatial dependency is introduced by a hidden Markov random field model. The posterior probability is estimated via likelihood-free approach, the Approximate Bayesian Computation. To improve the detection of chromatin interactions from existing methods assuming independence, HiC-ACT [99] performs a post-processing based on an aggregated Cauchy combination test (ACT).

Clustering-wise, cLoops [100] finds candidate loops by applying cDBSCAN to paired-end tags/reads, an optimized version of DBSCAN. A further optimization, known under the name of blockDBSCAN, was used in the second version of the tool: cLoops2 [101]. As discussed later, these are not the only methods relying on supervised-learning.

An example of a structural implementation is found in HOMER’s findTADsAndLoops [60], which is capable of simultaneously detecting both TADs and loops. Once relative contact maps are produced for each chromosome, HOMER analyzes them to find locally dense regions of contacts, which are then scored by their Hi-C interaction density normalized to the read depth.

Finally, a few methods offering supervised learning frameworks have been recently published. Peakachu [102] applies a hyperparameter search to find the best random forest model separating two classes: positive (any list of interactions) and negative (randomly sampled loci). LOOPbit [103] is a Convolutional Neural Network (CNN) trained to predict the location of loops. The network contains the following components: flattening of the input matrix, dense layer (with ReLu activation function), dropout, final dense layer (with Softmax activation function) that classifies the input into two different classes: loop and no-loop.

### Image representation

Not surprisingly, most approaches make extensive use of methodologies from computer vision and image processing.

Similarly to Chromosight [88], MUSTACHE [26] can be considered a template-based method. It makes use of convolutions: it normalizes the input contact map, convolves it with Gaussians of increasing scales – thus computing its Gaussian-kernel scale-space representation, and finally estimates the scaled normalized-Laplacian via the difference-of-Gaussian function. Candidate loops are found by comparing each pixel to its $3 \times 3 \times 3$ neighborhood, where the first 2D comes from the original image space while the last one originates with the convolution process. Additional filtering criteria are tested to remove false positives.

SIP [104] adopts image adjustment steps: Gaussian blur, contrast enhancement, white top-hat; it then proceeds by analyzing the image by sliding windows using a regional maxima detection algorithm to produce a preliminary list of candidate loops, which is then filtered by applying a set of criteria. Its working principles can be considered as inspired by structural pattern recognition.

Statistical pattern recognition comprises a few algorithms. A method using local statistics is Juicer’s HICCUPS [16] which examines each pixel in the Hi-C image by comparing its contact frequency to four kinds of local neighborhoods: (i) donut-shaped; (ii) lower-left; (iii) vertical and (iv) horizontal neighborhoods around the pixel of interest. Retrieved pixels are then grouped via a clustering-like method. An alternative implementation is available under the name HiCPeaks (https://pypi.org/project/hicpeaks/). Statistical modeling is performed in LASCA [105]. It starts by the fitting a Weibull distribution-based statistical background model to each diagonal of the input (corrected) Hi-C matrix; for every pixel, a $q$-value — i.e. a corrected $P$-value — that quantifies the probability of finding a corresponding model pixel with the same or higher intensity is computed; an user-defined threshold is used on $q$-values to find relevant pixels, which are then grouped into clusters; the cluster centers are further filtered according to their aggregate peak analysis and the surviving ones are returned. CHESS [80], named in Section TADs, can also identify points.

Learning-based pattern recognition includes both CHESS and Juicer’s HICCUPS, as they also make use of concepts from unsupervised learning. A neural architecture is presented under the name RefHiC [106]. It is based on two components: (i) a neural network — made up of an encoder, an attention module and a task-specific head — predicts loop scores for every candidate pair; (ii) a task-specific component selects one loop from each high-scoring cluster, where clusters are produced by density-based clustering.

## NAVIGATING THE METHODOLOGICAL MAZE

Approaches that utilize a matrix representation of Hi-C data do not typically introduce explicit geometric definitions of what a pattern is. Instead, they focus on mere numerical properties. In the case of compartments, sub-compartments, and TADs, squares and rectangles are identified a-posteriori by recognizing slices of matrix rows or columns with significant count variation (i.e. the candidate boundaries). Alternatively, rows/columns are grouped together based on some concept of similarity, often accompanied by statistical assumptions. Similarly, dots are defined by sets of matrix entries that are relatively close to each other in terms of matrix coordinates and have relatively high values. While this data representation allows to unlock a wide variety of algorithms from matrix theory (e.g. eigenvalue and SVDs), discarding geometric information can make parameter interpretability and result analysis challenging.

Algorithms that interpret Hi-C data as images leverage methodologies from computer vision, such as intensity transformations, spatial filtering and other image transforms. Patterns are discovered using families of templates, sliding windows, or segmentation techniques. The main advantage, compared to matrix-oriented approaches, is the higher interpretability and intuitivity. However, these algorithms can be significantly slower, and their performance is influenced by the size of the patterns being analyzed.

Considering graphs offers the advantage of representing Hi-C data in a higher-order form, allowing for the application of a rich set of algorithms and techniques from graph theory. However — similarly to matrix-based methods — visual interpretability is limited; algorithms are also less intuitive, which can be problematic for non-experts in the field. Constructing a graph for high-order adjacency matrices can be computationally demanding. Another drawback compared to image-based approaches is the potential loss of pixel-level information, especially when the graph representation is based on higher-level features or abstractions. It is worth noting that, with the sole exception of two algorithms, graph-based approaches have so far focused on TAD recognition.

When considering the various classes of algorithms in pattern recognition, template-based methods stand out for their high level of interpretability. These algorithms directly match patterns to predefined templates, making it easy to understand how the recognition process works. Moreover, template-based methods offer a straightforward generizability since new patterns can be recognized by creating new templates. However, these methods can perform poorly when patterns deviate significantly from the available templates.

On the other hand, structural pattern recognition algorithms provide the advantage of defining hierarchies of patterns. This capability becomes particularly valuable when dealing with TADs. While these algorithms offer the potential for more complex pattern relationships, they may trade off some efficiency compared to other methods.

Statistical methods naturally handle data uncertainty, accounting for factors such as noise, outliers and small variations in patterns. However, these methods rely on assumptions about the underlying data distribution. In real-world scenarios, these assumptions may not always hold true, leading to potential inaccuracies in recognition.

Finally, learning-based methodologies offer the flexibility of working without distribution assumptions. This advantage allows these algorithms to adapt to a wide range of patterns. However, this flexibility often comes at the expense of interpretability, as the inner workings of the model can be complex and challenging to understand. Neural architectures, a type of learning-based method, typically require rich benchmarks for effective training, which may not always be readily available.

## CONCLUSIONS AND PERSPECTIVES

The Hi-C technology has revolutionized the way we study the organization of chromatin in the nucleus, turning an inherently 3D environment into a 2D one. In this review, we have explored the core representations (matrix, image, graph) of Hi-C data and discussed how chromatin structures geometrically appear therein. Additionally, we have discussed the various computational methods within geometric pattern recognition (template-based, structural, statistical, learning-based) to automatically recognize such shapes. These algorithms range from simple clustering-based algorithms to more sophisticated techniques rooted in topological data analysis and machine learning. Although existing algorithms have provided valuable insights into the spatial organization of chromatin, they still face several challenges.

At present, a comprehensive framework for automatically identifying specific geometric shapes at various scales is lacking. For instance, an algorithm simultaneously identifying squares and rectangles representing TADs, compartments and SVs is currently lacking. This computational tool should also account for local signal variations and the combination of simple patterns, including single- or paired-rectangles with stronger signals at specific vertices.

Beyond the composition of simple shapes, one of the main obstacles is the automatic recognition of complex geometric patterns. Such patterns may arise from the overlapping of multiple types of simple shapes or from newly-discovered chromatin structures.

To truly understand complex systems and phenomena, we will need to integrate multiple data representations. A first step in this direction was recently taken with GILoop [90], a neural architecture synergizing the information of both image- and graph-interpretations of Hi-C data to recognize chromatin loops.

Genomic data are becoming increasingly detailed and accurate. Nevertheless, the exponential surge in the amount of information calls for the development of cutting-edge methods dedicated to Hi-C data analysis. For example, with recent ultra-deep Hi-C with resolutions at kilobase level or beyond, Hi-C matrices become extremely large, with billions to trillions of entries. As a result, working with such matrices using (most of) the tools mentioned in this review becomes impractical or intractable. Moreover, signal sparsity currently represents an almost insurmountable challenge to pattern recognition when low sequencing depths are used at such resolution [43]. The sparseness of measured interactions poses an analysis challenge to other C-based techniques, such as single-cell Hi-C (scHi-C) [107]; despite presenting obstacles, single-cell maps clearly reflect hallmarks of chromosomal organization and can therefore provide valuable insight into cell-to-cell variability. Beyond sparseness, noise and other forms of data perturbation continue to be a prominent topic in Hi-C data analysis, with recent research focusing on neural architectures [108, 109]; the use of results from the computer vision community, e.g. [110, 111] could further improve results.

A promising research direction involves the study of geometric patterns as dynamic entities, which provides significant information about the evolving nature of chromatin organization over time.

Finally, to truly advance the field, we need to establish solid definitions of what constitutes the different patterns seen in Hi-C data. Only then can computational methods be tuned to detect these efficiently and robustly. As for now, recognized patterns are validated for their biological relevance — without any gold-standard set to benchmark or test the methods — via experimental replicates to measure consistency or using synthetic data. This is, for example, the case for TAD callers, where CTCF motif instances and ChIP-seq signal are used [112]. As for the recently-introduced Pore-C technology [113], the task of introducing sound definitions that encompass interactions among more than two genomic loci becomes even more challenging.

**Key Points**

- This review addresses the correspondence between chromatin biology and geometry emerging from Hi-C data.
- Geometric pattern recognition is a powerful toolbox for understanding 3D genome organization.
- Existing algorithms are categorized on the basis of the data representation and paradigms they make use of.
- Despite the progress in the automatic recognition of geometric patterns, several challenges remain unresolved.

## Data Availability

Data sharing not applicable to this article as no datasets were generated or analysed during the current study.

## Author Biographies

**Andrea Raffo**, PhD, is a postdoctoral candidate in Bioinformatics at the University of Oslo, having obtained his PhD in Mathematics from the same institution in 2022. His research interests revolve around geometric modeling and shape analysis, with focus on various application domains such as biosciences, computer graphics and Computer-Aided Design.

**Jonas Paulsen**, PhD, is an Associate Professor at the Department of Biosciences at the University of Oslo. With a significant cross-disciplinary focus spanning computational and life sciences, his research involves the development of computational methods and analyses to understand the structure and function of genomes in 3D. He is an active member of the International Nucleome Consortium and a contributor to the 4DNucleome Program.
